# Effect of Nitrogen Application Rate on Growth Physiology, Yield Quality, and Nitrogen Fertilizer Utilization Rate of *Liriope muscari* in Pots

**DOI:** 10.3390/biology14081104

**Published:** 2025-08-21

**Authors:** Yuhong Yuan, Jihong Xiao, Shaoyan Liu, Tianyou He, Jundong Rong, Yushan Zheng

**Affiliations:** 1 Department of Economic Management, Fujian Forestry Vocational and Technical College, Nanping 353000, China; yuanyuhong1987@163.com (Y.Y.); 14759953146@163.com (S.L.); 2 College of Landscape Architecture and Art, Fujian Agriculture and Forestry University, Fuzhou 350002, China; xiaojihong1998@163.com (J.X.); hetianyou1985@163.com (T.H.); 3 College of Forestry, Fujian Agriculture and Forestry University, Fuzhou 350002, China; rongjd@126.com

**Keywords:** pot experiment, morphological development, photosynthetic physiology, yield and quality, nitrogen fertilizer utilization

## Abstract

*Liriope muscari* is a medicinal and ornamental herbaceous plant with significant economic value, as its tuberous roots are used for medicinal purposes. However, the current production of medicinal plants is characterized by wasteful use of resources and ecological risks caused by the unreasonable application of nitrogen fertilizers. In this study, based on uniform application of phosphorus and potassium fertilizers, six nitrogen application levels were set in pot experiments (expressed as N): N0: 0 kg/ha, N1: 208.33 kg/ha, N2: 416.66 kg/ha, N3: 625 kg/ha, N4: 833.33 kg/ha, N5: 1041.66 kg/ha). The morphological characteristics, photosynthetic physiology, tuber yield and quality, and seven nitrogen fertilizer utilization indices of *L. muscari* were analyzed and measured. Correlation analysis and structural equation modeling (SEM) were employed to investigate the mechanism by which nitrogen influences its growth and development, photosynthetic characteristics, tuber yield and quality, and nitrogen fertilizer utilization efficiency. The results showed that (1) nitrogen significantly promoted plant height, crown width, tiller number, and chlorophyll synthesis, with the N3 treatment (625 kg/ha) reaching the peak value, and the crown width and tiller number increasing by 26.44% and 38.90% compared to N0; the total chlorophyll content and net photosynthetic rate increased by 39.67% and 77.04%, respectively, compared to N0; high nitrogen (N5) inhibited photosynthesis and increased intercellular CO_2_ concentration; (2) Fresh weight of tuberous roots, polysaccharide content, and saponin C content peaked at N3 (34.67 g/plant, 39.89%, and 0.21%), respectively, representing increases of 128.69%, 28.37%, and 33.66% compared to N0; (3) Nitrogen uptake, nitrogen fertilizer utilization efficiency, agronomic utilization efficiency, and apparent utilization efficiency were optimal at N3, while high nitrogen (N4–N5) reduced nitrogen fertilizer efficiency by 40–60%; (4) SEM analysis indicated that tiller number and transpiration rate directly drive yield, while stomatal conductance regulates saponin C synthesis. Under the experimental conditions, 625 kg/ha is the optimal nitrogen application rate balancing yield, quality, and nitrogen efficiency. Excessive nitrogen application (>833 kg/ha) induces photosynthetic inhibition and “luxury absorption”, leading to source-sink imbalance and reduced accumulation of secondary metabolites. This study provides a theoretical basis and technical support for the precise management of nitrogen in Liriope-type medicinal plants. It is expected to alleviate the contradictions of “high input, low output, and heavy pollution” in traditional fertilization models.

## Simple Summary

Farmers growing *Liriope muscari*—an herb valued for its medicinal roots and ornamental use—often apply excessive nitrogen fertilizer. This practice wastes resources, pollutes waterways through fertilizer runoff, and ultimately limits crop yields. To determine the optimal nitrogen level, we grew plants in pots with six fertilizer treatments (0 to 1042 kg/ha) while maintaining constant levels of other nutrients. The results showed that moderate nitrogen application (625 kg/ha) maximized plant growth, increasing plant size by 26%, leaf production by 34%, and root yield by 129% compared to unfertilized plants. This treatment also enhanced photosynthesis by 77%, increased key medicinal compounds (saponin C by 28%, polysaccharides by 34%), and improved fertilizer efficiency by 19%. Our findings demonstrate that precisely managed nitrogen at 625 kg/ha allows farmers to achieve higher yields of better-quality medicinal roots while reducing environmental pollution and production costs. This strategy supports the sustainable cultivation of economically important medicinal plants.

## 1. Introduction

*Liriope muscari*, a perennial evergreen herbaceous plant of the Liliaceae family that has both excellent ornamental value [1,2,3] (such as flower border edging and riverbank slope protection) and important medicinal value. In 2010, it was included in the Chinese Pharmacopoeia under the entry for *Liriope*, and its dried tuberous roots have the effects of nourishing yin, generating fluids, moistening the lungs, clearing the heart, and calming the mind [4,5,6,7]. With the internationalization of traditional Chinese medicine and the growing demand for health and wellness, the cultivation of *L. muscari* has become an important economic industry. However, in pursuit of high yields, farmers often overuse nitrogen or use unreasonable fertilizer ratios. This not only increases production costs, but also leads to significant nitrogen losses (such as ammonia volatilization and nitrate nitrogen leaching), causing environmental problems such as water eutrophication and greenhouse gas emissions [8,9]. Therefore, optimizing the nitrogen fertilizer application model of *L. muscari* and improving its nitrogen use efficiency (NUE) are key to achieving synergistic improvements in the economic and ecological benefits of this industry.

Nitrogen is a key element regulating plant growth, development, yield, and quality formation. Different plants exhibit significant differences in nitrogen absorption, utilization, and response [10,11,12,13,14,15]. For medicinal herbaceous plants, nitrogen regulation is particularly critical, as it not only influences biomass accumulation but also profoundly affects the synthesis and accumulation of secondary metabolites, thereby determining the yield and quality of medicinal materials. Currently, most studies on *L. muscari* focus on germplasm resource evaluation [3,4], secondary metabolite fractionation and pharmacological activity mechanism analysis and identification [5,6,7,16], cultivation physiology [17,18], and molecular regulation basis [19,20], while core research on its nitrogen nutrition physiology is relatively scarce. In particular, it is unclear how nitrogen regulates photosynthetic carbon assimilation, nitrogen use efficiency, and ultimately yield and medicinal quality (such as the content of key active ingredients).

Based on this, this study proposes the core hypothesis that the morphological development, photosynthetic indicators, nitrogen use efficiency, yield, and medicinal qualities of *L. muscari* have specific response thresholds and patterns to nitrogen supply levels. To test this hypothesis, this study set up different nitrogen levels through pot experiments to investigate: (1) How do the photosynthetic characteristics and nitrogen use efficiency (NUE) of *L. muscari* respond to a gradient nitrogen supply? (2) What is the relationship between the tuber yield, medicinal quality, and key physiological indicators of *L. muscari* at different nitrogen levels? (3) What is the optimal nitrogen application rate to achieve synergistic improvement in *L. muscari* growth, tuber yield, medicinal quality, and nitrogen use efficiency? This study aims to elucidate the physiological mechanisms of *L. muscari*’s response to nitrogen and determine the appropriate nitrogen fertilizer application rate for high-yield, high-quality cultivation, providing a theoretical basis for “cost reduction and efficiency improvement” and green sustainable cultivation in actual production.

## 2. Materials and Methods

### 2.1. Materials

Test seedlings: Mainly cultivated varieties from the GAP construction demonstration base of *L. muscari* in Luoxi Town, Quanzhou City, Fujian Province. Before transplanting, seedlings were cultivated in an open-field nursery on a sandy loam soil substrate with the following properties: pH 6.28, organic matter 27.25 g·kg^1^, alkali-hydrolyzable N 49.26 mg/kg, available P 11.17 mg/kg, and exchangeable K 24.68 mg/kg. On 5 April 2022, vigorous 12-month-old seedlings (plant height: 8.2 ± 1.2 cm) with well-developed root systems and free of diseases/pests were selected and transplanted into 30 cm × 24 cm × 24 cm polyethylene pots.

Potting Substrate: Each pot was uniformly filled with approximately 7.0 kg of air-dried, homogenized sandy loam soil passed through a 2 mm sieve and thoroughly mixed. Physicochemical properties of the homogenized potting substrate (representing the 0–20 cm soil layer): Field capacity: 28.41%, pH: 6.08, organic matter content: 14.83 g/kg, available nitrogen content: 75.81 mg/kg, available phosphorus content: 4.96 mg/kg, and available potassium content: 17.58 mg/kg.

Experimental site overview: This experiment was conducted from April 2022 to April 2023 in the experimental greenhouse of the Forestry Department of Fujian Forestry Vocational and Technical College in Yanping District, Nanping City, Fujian Province (117°50′ E, 26°51′ N). The experimental site has a mild and humid subtropical monsoon climate, with an annual average temperature of 20.8 °C and an annual precipitation of 1847.0 mm, with rainfall primarily concentrated from March to June.

### 2.2. Experimental Design

The experiment was conducted from April 2022 to March of the following year at the Nursery Production Experimental Base of the Forestry Department of Fujian Forestry Vocational and Technical College. A randomized complete block design (RCBD) was implemented with three blocks. Each block contained 10 pots of *L. muscari*, with three uniform and vigorous seedlings transplanted per pot at 50 cm pot spacing. Based on preliminary surveys of fertilization practices among growers in Fujian’s main production regions, six nitrogen gradients ranging from conventional farming rates to excessive application levels were established: N0 (0 g/pot, equivalent to 0 kg/ha), N1 (1.5 g/pot, equivalent to 208.33 kg/ha), N2 (3.0 g/pot, equivalent to 416.66 kg/ha), N3 (4.5 g/pot, equivalent to 625.00 kg/ha), N4 (6.0 g/pot, equivalent to 833.33 kg/ha), and N5 (7.5 g/pot, equivalent to 1041.66 kg/ha). Phosphorus and potassium fertilizers were applied as basal fertilizers in a single application, with application rates of 277.77 kg/ha and 208.33 kg/ha, respectively. Standard management practices were adopted throughout the trial period, including regular watering, soil loosening, weed control, and pest management.

Test fertilizers: ordinary urea (N content ≥ 46.0%), calcium superphosphate (P_2_O_5_ ≥ 12%), and potassium sulfate (K_2_O ≥ 51%), all purchased from Hubei Fengle Eco-Fertilizer Co., Ltd. (Jingmen, China).

### 2.3. Measurement Content and Methods

During the tillering period (July), the swelling period (September), the dormancy period (November), and the harvest period (March of the following year), the growth indicators and chlorophyll content of *L. muscari* were investigated, including plant height, crown width, number of tillers, and number of leaves (Table A1 of the Appendix A). Chlorophyll content was determined using the ethanol extraction colorimetric method (Table A2 of the Appendix B).

On 18 September 2022 (the swelling period), 9:00–11:00 a.m., clear and sunny, gas exchange parameters were measured under natural light conditions using a LI-COR 6400XT portable photosynthesis meter (LI-COR Biosciences, Lincoln, NE, USA) [21], with CO_2_ concentration set at 400 μmol·mol^−1^, gas flow rate at 500 μmol·s^−1^, and leaf temperature at 28 ± 1 °C. Three representative seedlings were selected for each treatment, and their fully expanded upper leaves facing the sun were measured. After the readings stabilized, net photosynthetic rate (Pn), intercellular CO_2_ concentration (Ci), stomatal conductance (Gs), and transpiration rate (Tr) were measured.

On 19–20 March of the following year (harvest period), the yield of individual tubers, saponin C, and polysaccharide content were measured. The saponin C content was measured using an extraction process established by the research team. Quantitative analysis was performed using high-performance liquid chromatography (HPLC) [22], employing a SinoChrom ODS-BP chromatographic column (5 μm, 150 mm × 4.6 mm). The mobile phase consisted of acetonitrile-water solution (52:48), with a flow rate of 1.0 mL/min, column temperature of 30 °C, and detection wavelength of 203 nm. A standard curve was plotted using saponin C standard (purity ≥ 98%) for quantification, with each sample analyzed in triplicate. And the polysaccharide content was measured using the phenol-sulfuric acid method [23], with absorbance measured at 490 nm using a UV–visible spectrophotometer (UV-9000S, Shanghai Yuanxi Instrument Co., Ltd., Shanghai, China). A standard curve was plotted using a glucose standard (purity ≥ 99%), with each sample analyzed in triplicate. Oven-dried plant samples were ground and sieved through a 60-mesh screen, and nitrogen content was determined using the semi-micro Kjeldahl method [24]. Six plants were randomly sampled from each treatment, with three replicates. Measured values for photosynthetic parameters, tuber yield, saponin C, polysaccharide, and nitrogen content are provided in Table A3 of the Appendix C. The formulas for nitrogen accumulation and utilization efficiency are as follows:

Nitrogen accumulation per plant = dry weight of the plant × nitrogen concentration

Nitrogen harvest index per plant = (nitrogen absorption by the root tuber)/nitrogen accumulation by the plant

Nitrogen fertilizer-utilization rate = (nitrogen accumulation by plants in the nitrogen-applied treatment − nitrogen accumulation by plants in the non-nitrogen-applied treatment)/total nitrogen applied × 100%

Nitrogen fertilizer agronomic utilization rate = (root yield in the nitrogen-applied treatment − root yield in the non-nitrogen-applied treatment)/total nitrogen applied × 100%

Nitrogen fertilizer-specific productivity = root yield/total nitrogen applied

Nitrogen fertilizer-physiological utilization rate = (nitrogen-applied treatment tuber yield − non-nitrogen-applied treatment tuber yield)/(nitrogen-applied treatment nitrogen uptake − non-nitrogen-applied treatment nitrogen uptake)

Nitrogen fertilizer-apparent utilization rate = (nitrogen-applied treatment plant nitrogen uptake − non-nitrogen-applied treatment plant nitrogen uptake)/nitrogen application rate × 100%

### 2.4. Data Analysis

Excel 2022 (Microsoft Corporation) software was used for data compilation, and R 4.3.2 software [25] was used for data processing. To investigate the effect of nitrogen on various indicators of *L. muscari*, the agricolae v1.3-6 [26] package was used to perform analysis of variance and LSD (Least Significant Difference) tests, and the ggplot2 v3.4.4 [27] package was used for graphing. To investigate the correlation between calorific value and various indicators, Pearson correlation analysis plots were created using the dplyr v1.1.3 [28], linkET v0.0.7 [29], and ggplot2 v3.4.4 [27] packages. To further investigate how the various indicators collectively influence calorific value, the readr v2.1.4 [30], corrplot v0.92 [31], and Hmisc v5.1-0 [32] packages were used to plot calorific heatmap diagrams. Based on the results of the Pearson correlation test, we conducted multicollinearity analysis to identify key indicators, and path analysis models were constructed using the tidyverse v2.0.0 [33], lavaan v0.6-16 [34], and semTools v0.5-6 [35] packages.

## 3. Results

### 3.1. Effects of Different Nitrogen Application Rates on the Morphological Development of Potted L. muscari

**Figure 1:**
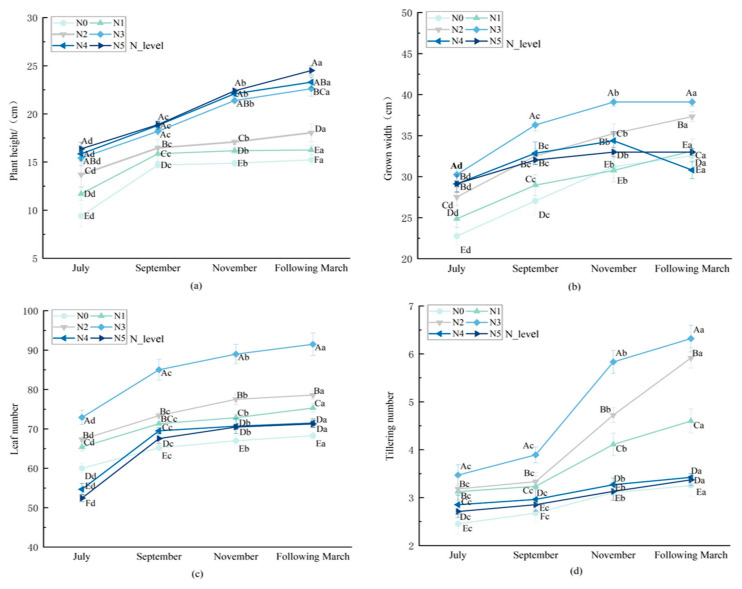
Effect of nitrogen application rate on morphological development indicators of potted *L. muscari.* Note: (**a**): plant height, (**b**): crown width, (**c**): leaf number, and (**d**): tiller number. Uppercase letters indicate significant differences between different nitrogen fertilizer treatments (*p* < 0.05), while lowercase letters indicate significant differences between different monthly treatments (*p* < 0.05). The same below.

As shown in Figure 1, nitrogen application rate and growth period significantly affect (*p* < 0.05) the growth indicators of *L. muscari*, and there was a significant interaction (*p* < 0.01). From July to November, the plant height, crown width, number of leaves, and number of tillers in each treatment group increased significantly (*p* < 0.05). With increasing nitrogen application, the plant height of *L. muscari* rose significantly (*p* < 0.05), while the crown width, number of leaves, and number of tillers exhibited an overall trend of first increasing and then decreasing. The crown width, number of leaves, and number of tillers reached their maximum values in N3. At the harvest period (March of the following year), the plant height, crown width, leaf number, and tiller number in the N3 treatment attained maximum values. At harvest (March of the following year), the plant height, crown width, leaf number, and tiller number in N3 were significantly increased by 41.96%, 26.44%, 33.97%, and 38.90%. Respectively, compared to N0. N4 and N5 treatments exhibited growth inhibition, with the number of leaves decreasing by 20.21% and 33.69%, respectively, compared to the control N0, and the number of tillers decreasing by 14.52% and 23.56%. This may be due to excessive nitrogen application, causing an imbalance in carbon–nitrogen metabolism, thereby inhibiting the accumulation of structural carbohydrates.

### 3.2. Effects of Different Nitrogen Application Rates on Photosynthetic Pigments in Potted L. muscari

**Figure 2:**
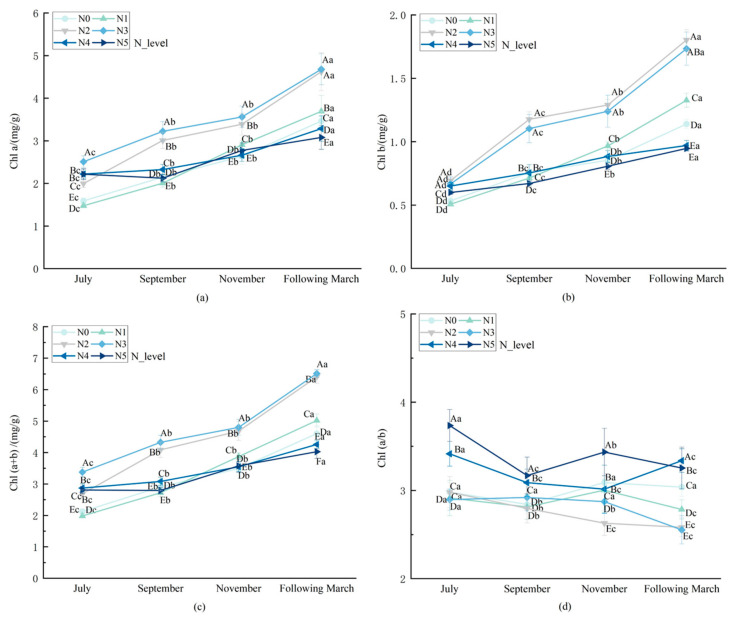
Effect of nitrogen application rate on photosynthetic pigment indices of potted *L. muscari.* Note: (**a**): chlorophyll a, (**b**): chlorophyll b, (**c**): chlorophyll (a + b), and (**d**): chlorophyll (a/b).

As shown in Figure 2, nitrogen application rate and growth stage had significant effects (*p* < 0.05) on chlorophyll content in *L. muscari.* The chlorophyll content of *L. muscari* increased gradually with the growth period, and the Chl (a/b) ratio ranged from 2.629 to 3.736, indicating a high ratio. With the increase in nitrogen application, the Chl a, Chl b, and Chl (a + b) contents showed a single-peak curve trend. In each growth period, the peak values of Chl a, Chl (a + b) reached their peak values at N3 (625.00 kg/ha), while Chl b peaked at N2 (416.66 kg/ha). At harvest, Chl a and Chl (a + b) in N3 were 35.24% and 39.67% higher than in N0, respectively, and the Chlb content in N2 increased by 58.71% compared to N0. The Chl (a/b) ratio showed a decreasing trend in the N0–N3 nitrogen application range and rebounded to 3.388 (*p* < 0.05) in the high nitrogen level range of N3–N5. It is worth noting that, compared with Chl a, Chl b reaches its peak at lower nitrogen levels, which may reflect that under low-to-medium nitrogen conditions (N1–N2), plants enhance light energy capture efficiency by increasing Chl b synthesis (the main component of LHCII) to compensate for the reduction in photosynthetic units under nitrogen limitation.

### 3.3. Effects of Different Nitrogen Application Rates on Photosynthetic Gas Parameters of Potted L. muscari

**Figure 3:**
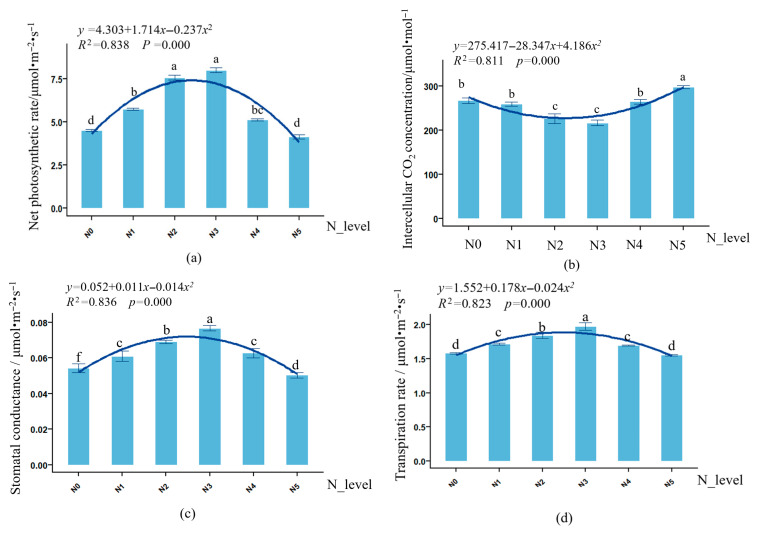
Effect of nitrogen application rate on photosynthetic parameters of potted *L. muscari*. Note: (**a**): net photosynthetic rate, (**b**): intercellular CO_2_ concentration, (**c**): stomatal conductance, and (**d**): transpiration rate. Integers between 0 and 5 correspond to N0−N5, which are used for curve fitting; different lowercase letters indicate significant differences between treatments (*p* < 0.05). The same below.

As shown in Figure 3, there was a highly significant (*p* < 0.001) quadratic relationship between nitrogen application rate and photosynthetic performance of *L. muscari* leaves. With increasing nitrogen application, the net photosynthetic rate, stomatal conductance, and transpiration rate of *L. muscari* leaves showed a trend of first increasing and then decreasing, reaching their maximum values in N3, which were 77.04%, 42.59%, and 24.94% higher than in N0, respectively. The intercellular CO_2_ concentration showed a trend of first decreasing and then increasing, reaching its minimum value in N3 and subsequently rebounding to a maximum value of 297.8 μmol·mol^−1^ (*p* < 0.05) in N5, representing an increase of 11.43% compared to N0, this may be due to high nitrogen inducing partial closure of stomata, reducing CO_2_ supply.

### 3.4. Effects of Different Nitrogen Application Rates on the Yield and Quality of Potted L. muscari

**Figure 4:**
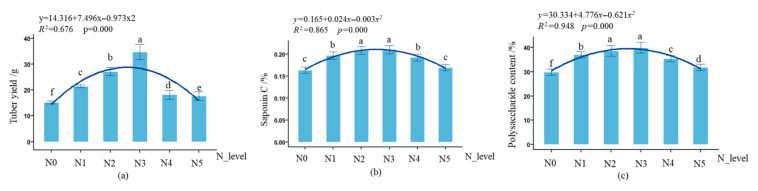
Effect of nitrogen application rate on the yield and quality of potted *L. muscari*. Note:(**a**): tuber yield, (**b**): saponin C, and (**c**): polysaccharide content.

As shown in Figure 4, the nitrogen application rate had a significant effect (*p* < 0.001) on the biomass accumulation and secondary metabolite synthesis of *L. muscari* rhizomes. With increasing nitrogen application rate, the yield of single rhizomes, polysaccharide content, and saponin C content exhibited a single-peak trend, first increasing and then decreasing, reaching a peak under the N3 treatment. The fresh weight of individual tuberous roots increased significantly by 128.69% from N0 to N3 (*p* < 0.001), while the N4–N5 treatments returned to the N0 level (*p* > 0.05); Polysaccharide content reached a peak at N3, increasing by 28.37% compared to N0 (*p* < 0.01); Saponin C content: N3 treatment was significantly higher than N0 (*p* < 0.05), but there were no significant differences between N2–N5 treatments (*p* > 0.05).

### 3.5. Effects of Different Nitrogen Application Rates on Nitrogen Fertilizer Utilization Efficiency of Potted L. muscari

Table 1 shows that the nitrogen application rate had a significant effect (*p* < 0.05) on nitrogen absorption and nitrogen fertilizer utilization rate in *L. muscari*. The nitrogen uptake of each nitrogen treatment was significantly higher than that of N0, with N3 reaching the peak value, which was 182.14% higher than N0. The nitrogen fertilizer utilization rate, agronomic utilization rate, and apparent utilization rate of *L. muscari* showed a trend of first increasing and then decreasing with increasing nitrogen application, reaching the highest value in N3, compared with the low-nitrogen N1 treatment, there was a significant increase (*p* < 0.05) of 34.02%, 4.74%, and 14.19%, respectively. In addition, the nitrogen fertilizer productivity and physiological utilization rate of *L. muscari* showed a continuous decreasing trend (*p* < 0.01) with increasing nitrogen application, with N1 having relatively high nitrogen fertilizer productivity and physiological efficiency rate, and N2 and N3 showed no significant difference in partial productivity and physiological efficiency.

### 3.6. Direct and Indirect Mechanisms by Which Various Indicators Affect the Yield and Quality of L. muscari Rhizomes

#### 3.6.1. Correlation Between Various Indicator Traits and Rhizome Yield and Quality

**Figure 5:**
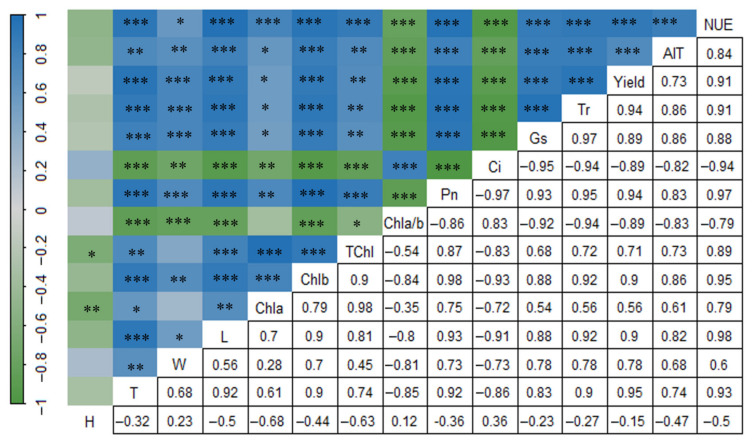
The Pearson correlation among the indicators. Note: H: plant height; T: tiller number; W: crown width; L: leaf number; Chla: chlorophyll a; Chlb: chlorophyll b; Tchl: chlorophyll (a + b); Chla/b: chlorophyll a/b ratio; Pn: net photosynthetic rate; Ci: intercellular carbon dioxide concentration; Gs: stomatal conductance; Tr: transpiration rate; Yield: tuber yield; AIT: saponin C; NUE: nitrogen fertilizer utilization rate. *: *p* < 0.05, **: *p* < 0.01, ***: *p* < 0.001.

Correlation analysis (Figure 5) showed that the yield, quality, and nitrogen fertilizer utilization rate of *L. muscari* were significantly positively correlated with T, W, L, Chla, Chlb, TChl, Pn, Tr, and Gs (*p* < 0.05) and significantly negatively correlated with Chla/b and Ci (*p* < 0.001). T, W, and L were significantly positively correlated with Chlb, Pn, Tr, and Gs (*p* < 0.01) and significantly negatively correlated with Chlb and Ci (*p* < 0.01). W was not correlated with Chla and Tchl. Chla/b was significantly positively correlated with T, W, L, Tr, and Gs (*p* < 0.01) and significantly negatively correlated with Chlb and Ci (*p* < 0.01). W was not correlated with Chla and Tch Gs (*p* < 0.01), and significantly negatively correlated with Chlb and Ci (*p* < 0.01). W was not correlated with Chla and Tchl. Chla/b was significantly negatively correlated with T, W, L, Chlb, Tchl, Pn, Tr, and Gs (*p* < 0.05), and significantly positively correlated with Ci (*p* < 0.001). Ci was significantly negatively correlated with T, W, L, Chla, Chlb, Tchl, Pn, Tr, and Gs (*p* < 0.01); H was significantly negatively correlated with Chla and Tchl (*p* < 0.05) and showed no significant correlation with yield, AIT, and NUE.

#### 3.6.2. Direct and Indirect Effects of Various Indicator Traits on Tuber Yield and Quality

**Figure 6:**
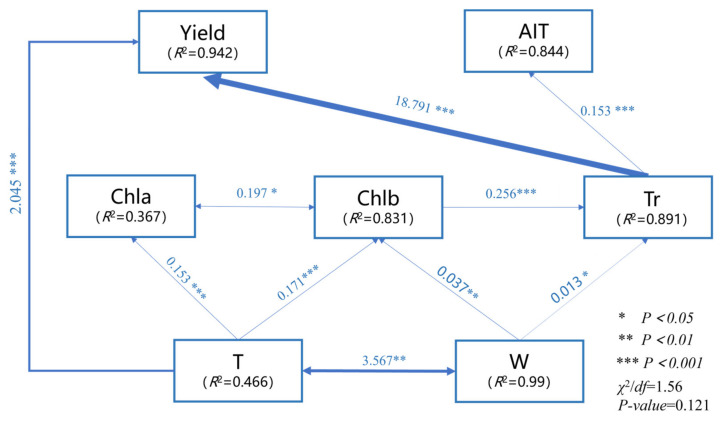
Structural equation model of various indicators and tuber yield and quality. Note: The thickness of the arrows indicates the strength of the relationship, and the values associated with the arrows indicate the standardized path coefficients.

Based on the results of the correlation analysis, trait indicators with significant effects on tuber yield and quality were selected, and indicators with strong collinearity were excluded. A structural equation model was then constructed (Figure 6). Model 1 < *χ^2^*/df = 1.56 < 3, *p* = 0.121 > 0.05, CFI = 0.996 > 0.95, RMSEA = 0.071 < 0.08, indicating that the model fits the data well. SEM indicates that T and Tr had direct positive effects on tuber yield (β = 2.045 and 18.791, respectively); Gs had a direct positive effect on tuber AIT (β = 0.153); Chlb and W exerted indirect effects via Tr (β = 0.256 and 0.013, respectively), indirectly influence tuber yield and AIT; T and W were correlated (β = 3.567), and Chla and Chlb were correlated (β = 0.197). Tr was the most significant factor influencing tuber yield and AIT, and it interacts with other factors to influence tuber yield and quality.

## 4. Discussion

### 4.1. Effect of Nitrogen on Growth, Physiological Traits, Yield, Quality, and Nitrogen Use Efficiency of L. muscari

The nitrogen supply level significantly regulates the morphological development, photosynthetic physiology, yield quality, and nitrogen fertilizer utilization of *L. muscari* in a dose-dependent manner. At the morphological level (Figure 1), at low nitrogen gradients (N0–N2), it promotes leaf expansion and stem cell differentiation [36]. At medium nitrogen levels (N3), source-sink balance is achieved through synergistic optimization of canopy structure; at high nitrogen levels (N4–N5), excessive stem and leaf growth occurs, with morphological variations exhibiting organ specificity, indicating that plant height growth exhibits a saturation effect in response to nitrogen, and beyond a certain nitrogen level, plant height growth becomes insignificant. The decrease in tiller number and leaf number may be due to plants adjusting morphological characteristics under high nitrogen stress to optimize resource allocation, reduce nitrogen consumption, and thereby improve nitrogen use efficiency [37,38]. Notably, tiller number is more sensitive to nitrogen than plant height, providing a specific indicator for morphological diagnosis of clump-forming medicinal plants.

At the level of photosynthetic pigments, this study found that the photosynthetic pigments of *L. muscari* exhibit hierarchical sensitivity to nitrogen: the characteristic pigment chlorophyll b of light-harvesting complex II (LHCII) reaches its peak in N2 (Figure 2b), while the reaction center pigments chlorophyll a and total chlorophyll achieve maximum accumulation in the N3 treatment (Figure 2a,c). This temporal difference suggests that plants prioritize LHCII synthesis under nitrogen-limited conditions to maintain basic light energy capture capacity [39,40]. Notably, total chlorophyll content in the high-nitrogen N5 treatment was significantly reduced by 37.21% compared to N3 (*p <* 0.05), but the chlorophyll (a/b) ratio significantly increased by 17.03% (*p <* 0.05), which may be attributed to ammonia toxicity caused by nitrogen metabolic imbalance, which disrupts pigment stability [41].

At the level of photosynthetic gas parameters, when nitrogen supply is adequate, the activity of Rubisco in plants is higher, leaf stomatal conductance is greater, and CO_2_ fixation capacity is enhanced, thereby reducing intercellular CO_2_ concentration and increasing photosynthetic rate [42,43]. The results of this study support this view. Notably, photosynthesis was significantly inhibited in the high-nitrogen N5 treatment. This phenomenon may involve multiple limiting factors: first, excessive nitrogen may disrupt hormonal balance within plants, reduce Rubisco enzyme activity, and weaken CO_2_ fixation capacity, thereby limiting photosynthesis [44]; second, excessive nitrogen application may damage chloroplast membrane structure, reduce chlorophyll content, and decrease photosynthetic efficiency [45]; third, high nitrogen treatment may increase photorespiratory carbon loss, consuming the assimilates produced by photosynthesis and reducing net photosynthetic rate [46].

In terms of tuber yield, quality, and resource utilization, nitrogen indirectly influences tuber yield, quality, and resource utilization efficiency by regulating the accumulation and distribution of dry matter [47]. We found that tuber yield and quality exhibit a significant single-peak response to nitrogen (Figure 4), which may essentially result from a three-tiered interactive effect involving photosynthetic productivity, assimilate allocation, and storage capacity activity [48]. Moderate nitrogen (N3) treatment first establishes an efficient photosynthetic source by aligning the canopy peak and net photosynthetic rate peak, and adequate nitrogen supply promotes plant synthesis of phenylalanine ammonia-lyase (PAL), thereby facilitating carbon flow toward tuber dry matter and secondary metabolite accumulation. High nitrogen (N5) treatment causes excessive growth of nutrient organs, forming a “metabolic sink limitation” [49], where excessive dry matter is allocated to stems and leaves, competitively depriving tuber enlargement of carbon substrates required, leading to reduced tuber yield. Additionally, excessive nitrogen may reduce starch content in tubers, resulting in decreased quality [50]. Changes in nitrogen fertilizer utilization efficiency further corroborate the aforementioned mechanisms (Table 1). N3 achieved synergistic peaks in total nitrogen absorption, agronomic nitrogen fertilizer utilization rate, apparent nitrogen fertilizer utilization rate, and physiological nitrogen utilization rate, while the high-nitrogen treatments (N4–N5) showed simultaneous declines in all four indicators. This phenomenon indicates that when nitrogen supply is excessive, root absorption capacity becomes saturated, leading to “luxury absorption”, where excess nitrogen accumulates in the vacuole in an inorganic state and fails to be converted into functional proteins. Simultaneously, excessive nitrogen disrupts plant nitrogen metabolism and secondary metabolism, thereby affecting carbohydrate accumulation and triggering the “high nitrogen, low-efficiency paradox” [51]. The study also found that the partial productivity of nitrogen fertilizer decreases continuously with increasing nitrogen application rates, consistent with the law of diminishing marginal returns [52].

### 4.2. Effect of Growth and Physiological Traits on Yield and Quality of L. muscari

According to the results of Pearson correlation analysis, tiller number, crown width, and leaf number, as indicators of plant morphology, showed significant positive correlations (*p <* 0.01) with photosynthetic pigments, photosynthetic gas parameters, and tuber yield and quality (Figure 5). The primary reason is that increases in tiller number, crown width, and leaf number significantly expand the light-interception area, enhance photosynthesis, accelerate metabolism, and increase Rubisco enzyme activity, increasing assimilated carbohydrates per unit time. At the same time, it promotes the partitioning of assimilates such as sucrose and starch to underground storage organs, thereby achieving the accumulation of secondary metabolites in tubers and an increase in the proportion of dry matter [53]. Therefore, the trends in the various indicators of *L. muscari* are the same. The results of path analysis (Figure 6) also demonstrate this: T and W significantly influence tuber yield and quality through both direct and indirect pathways. T directly drives an increase in leaf number (β = 2.045, *p* < 0.001), enhancing “source” capacity to promote assimilate accumulation and thereby directly increasing yield; W has direct effects on Chlb (β = 0.017, *p* < 0.01) and Tr (β = 0.013, *p* < 0.05), indirectly enhancing yield by expanding leaf area to enhance light energy capture and promote water-nutrient transport. Transpiration rate is the most important factor influencing yield and quality(β = 18.791, *p* < 0.001). Transpiration rate is a key indicator of the dynamic balance of water absorption, transport, and loss in plants, driving the upward transport of water and mineral nutrients through transpiration pull, providing the material basis for plant growth and metabolism [54]; higher transpiration rate ensures CO_2_ supply through stomatal opening, maintains a high photosynthesis rate, and provides carbon sources and energy for yield formation and saponin synthesis [55], ultimately significantly improving the economic quality of *L. muscari*. There is a significant positive correlation between tiller number and crown width *(p <* 0.01). *L. muscari* is a typical clump-forming perennial herbaceous plant. An increase in T increases photosynthetic functional units, directly increasing the total amount of photosynthetic pigments and enhancing the plant’s overall photosynthetic “source” capacity [56], thereby indirectly enhancing photosynthesis; the expansion of crown width facilitates the faster dissipation of water vapor produced by leaf transpiration and CO_2_ consumed by photosynthesis, creating a favorable microenvironment for leaf gas exchange. The two are in a potential positive feedback loop. Therefore, plants with larger crown widths and more tillers typically possess stronger photosynthetic production potential, which is a crucial guarantee for biomass accumulation and yield formation.

## 5. Conclusions

This study revealed the response patterns and internal mechanisms of potted *L. muscari* growth, development, and quality formation to nitrogen nutrition. Nitrogen supply significantly affects the carbon–nitrogen metabolic balance. The N3 (625 kg/ha) treatment is the theoretically optimal nitrogen application rate. At this dose, *L. muscari* achieves simultaneous increases in tuber yield and active ingredient content through synergistic optimization of canopy structure, photosynthetic efficiency, and nitrogen assimilation capacity. Excessive nitrogen application (>833 kg/ha) leads to excessive growth of vegetative organs, photosynthetic inhibition, and imbalance between nitrogen sources and sinks, resulting in reduced tuber yield and quality, while also causing “excessive nitrogen absorption” that lowers utilization efficiency. The current experimental results are based on a controlled pot culture environment and can provide a theoretical reference for field trials. However, further verification is needed in actual large-scale field applications to investigate the interactions between soil fertility, precipitation conditions, and population density with individual competition. For future field trials, it is recommended to combine real-time monitoring of nitrogen demand with tillering dynamics and canopy expansion rates. The nitrogen level gradient design should be based on the optimal nitrogen amount in potted plants (625 kg/ha), with a ±20% floating range.

## Figures and Tables

**Table 1:** Effects of nitrogen application on nitrogen use efficiency of potted *L. muscari.*

Treatment	NU	ANUE	NUE	FNUE	PNUE	NUI
	g/Plant	g g^−1^	%	%	%	%
N0	0.26 ± 0.04 e	—	—	—	—	—
N1	0.38 ± 0.03 d	23.23 ± 0.06 a	14.05 ± 1.41 c	6.75 ± 0.09 b	11.49 ± 1.01 c	48.04 ± 1.18 a
N2	0.57 ± 0.12 b	14.74 ± 0.03 b	17.01 ± 1.50 b	6.50 ± 0.14 b	12.96 ± 0.91 b	38.24 ± 1.11 b
N3	0.78 ± 0.16 a	12.56 ± 0.12 bc	18.83 ± 0.85 a	7.07 ± 0.15 a	13.12 ± 0.51 a	37.5 ± 1.68 b
N4	0.58 ± 0.21 c	4.94 ± 0.04 d	8.90 ± 0.92 d	0.82 ± 0.03 d	8.92 ± 0.81 d	9.17 ± 1.85 c
N5	0.57 ± 0.12 c	3.84 ± 0.08 e	6.71 ± 0.42 e	0.54 ± 0.01 d	6.81 ± 0.21 e	8.10 ± 1.50 d

Note: NU: nitrogen uptake; ANUE: nitrogen fertilizer productivity; NUE: nitrogen fertilizer utilization rate; FNUE: nitrogen fertilizer agronomic utilization rate; PNUE: nitrogen fertilizer apparent utilization rate; NUI: Nitrogen fertilizer physiological utilization rate; different lowercase letters indicate significant differences between treatments (*p* < 0.05).

## Data Availability

The original contributions presented in this study are included in the article. Further inquiries can be directed to the first author.

## Appendix A

**Table A1:** Raw data on morphological indicators of potted *L. muscari*.

N-Level	H				T				W				L			
	Jul	Sep	Nov	Mar	Jul	Sep	Nov	Mar	Jul	Sep	Nov	Mar	Jul	Sep	Nov	Mar
N0	10.4	14.9	15.2	15.60	3.00	3.15	3.36	3.78	23.10	28.30	31.50	31.90	60.00	63.90	68.40	69.24
N0	9.69	14.32	14.88	15.37	2.75	2.86	3.29	3.33	24.66	29.59	32.50	32.60	58.80	62.48	66.21	67.87
N0	8.11	14.88	14.57	14.71	3.20	3.22	3.26	3.55	20.52	26.24	29.67	30.12	61.20	63.21	66.37	67.76
N1	11.4	15.2	16.5	16.80	2.94	3.15	4.24	5.04	23.30	27.70	29.50	31.70	65.20	70.80	72.13	74.20
N1	12.52	14.65	16.15	15.93	3.21	3.34	3.84	3.51	24.35	26.83	30.51	30.89	66.51	71.46	73.56	75.50
N1	11.17	16.24	15.91	16.04	3.23	3.12	4.25	5.25	26.96	29.36	32.29	33.80	64.50	71.25	72.85	76.23
N2	14.60	16.21	17.4	18.70	3.22	2.94	4.83	6.06	28.80	31.60	32.80	36.90	68.00	72.50	75.60	79.80
N2	12.17	15.76	17.03	18.50	2.69	3.17	4.33	5.56	26.84	32.74	36.91	37.95	66.90	73.99	74.13	77.88
N2	14.31	17.44	16.84	16.95	3.34	3.89	5.01	6.13	26.96	33.25	36.07	37.15	67.12	73.63	76.81	78.12
N3	15.8	18.5	22.1	22.67	3.68	3.94	6.93	7.40	30.10	37.00	38.60	38.80	71.60	87.40	91.00	95.80
N3	14.28	18.32	20.79	24.03	3.25	3.71	6.56	7.01	29.55	36.40	39.61	39.72	68.88	82.21	86.25	88.41
N3	15.96	17.76	20.97	21.14	3.48	4.03	7.01	7.55	31.12	35.53	39.08	41.13	72.30	85.45	89.72	90.25
N4	16.05	19.11	21.43	22.9	1.61	1.89	2.00	2.51	23.30	34.40	34.60	34.70	33.40	41.40	49.60	52.60
N4	16.20	18.89	20.97	23.04	1.67	1.86	2.34	2.68	22.11	32.43	36.59	36.04	36.27	39.90	53.23	56.64
N4	15.57	18.34	20.92	23.97	1.85	2.11	2.57	2.96	23.96	31.76	34.94	36.65	34.33	40.25	52.26	54.23
N5	16.29	19.20	21.70	24.12	1.68	2.21	2.73	3.00	23.40	31.50	32.10	31.70	31.40	40.20	43.00	44.40
N5	15.73	18.58	21.25	24.34	2.52	2.62	3.01	3.05	21.96	32.64	34.61	34.71	32.77	39.52	43.25	45.21
N5	17.10	18.87	21.26	25.08	1.36	2.56	2.87	3.02	24.08	31.95	32.27	33.84	33.12	41.94	44.19	46.23

## Appendix B

**Table A2:** Raw data on photosynthetic pigment indices of potted *L. muscari*.

N-Level	Chla				Chb				Tchl				ChL (a/b)			
	Jul	Sep	Nov	Mar	Jul	Sep	Nov	Mar	Jul	Sep	Nov	Mar	Jul	Sep	Nov	Mar
N0	1.60	2.15	2.67	3.34	0.51	0.71	0.83	1.11	2.11	2.86	3.50	4.44	3.11	3.03	3.22	3.01
N0	1.65	2.26	2.73	3.56	0.57	0.66	0.85	1.18	2.21	2.92	3.58	4.74	2.89	3.40	3.20	3.01
N0	1.52	2.04	2.54	3.48	0.52	0.62	0.88	1.13	2.03	2.66	3.41	4.61	2.94	3.30	2.89	3.08
N1	1.39	2.01	2.91	3.69	0.51	0.68	0.99	1.33	1.90	2.68	3.90	5.02	2.73	2.98	2.95	2.78
N1	1.42	1.97	2.99	3.85	0.49	0.75	0.99	1.34	1.91	2.72	3.98	5.19	2.91	2.64	3.01	2.87
N1	1.32	1.91	2.84	3.53	0.51	0.71	0.92	1.31	1.84	2.62	3.76	4.84	2.58	2.69	3.08	2.69
N2	2.00	3.09	3.23	4.61	0.69	1.11	1.26	1.80	2.69	4.20	4.49	6.40	2.90	2.78	2.56	2.56
N2	2.09	3.01	3.44	4.86	0.68	1.21	1.30	1.81	2.77	4.22	4.74	6.67	3.09	2.48	2.65	2.68
N2	1.90	2.93	3.47	4.38	0.71	1.20	1.31	1.80	2.61	4.13	4.78	6.17	2.66	2.44	2.65	2.43
N3	2.50	3.23	3.66	4.61	0.67	1.09	1.27	1.73	3.18	4.32	4.93	6.34	3.73	2.97	2.88	2.66
N3	2.65	3.06	3.52	4.96	0.67	1.11	1.22	1.82	3.32	4.17	4.74	6.78	3.93	2.75	2.88	2.73
N3	2.38	3.37	3.51	4.47	0.65	1.11	1.23	1.65	3.03	4.48	4.74	6.12	3.65	3.02	2.86	2.71
N4	2.22	2.32	2.66	3.27	0.65	0.75	0.88	0.97	2.87	3.07	3.54	4.23	3.41	3.11	3.01	3.39
N4	2.33	2.45	2.81	3.49	0.64	0.77	0.86	0.91	2.98	3.22	3.67	4.39	3.62	3.17	3.26	3.85
N4	2.11	2.21	2.52	3.11	0.66	0.74	0.90	1.04	2.77	2.95	3.43	4.15	3.21	2.99	2.79	2.98
N5	2.21	2.22	2.76	3.13	0.60	0.67	0.84	0.91	2.81	2.89	3.60	4.04	3.71	3.31	3.29	3.42
N5	2.32	2.33	2.93	3.09	0.57	0.69	0.76	0.94	2.89	3.02	3.69	4.03	4.04	3.40	3.83	3.28
N5	2.10	2.13	2.62	3.02	0.63	0.66	0.82	0.98	2.73	2.79	3.44	4.00	3.36	3.24	3.22	3.08

## Appendix C

**Table A3:** Raw data on photosynthetic gas parameters, tuber yield, and quality of potted *L. muscari*.

N-Level	Pn	Ci	Gs	Tr	Yield	AIT	PC	NU
N0	4.52	261.04	0.057	1.592	15.11	0.16	30.12	0.258
N0	4.45	266.53	0.054	1.581	15.16	0.16	29.92	0.253
N0	4.57	274.26	0.052	1.568	15.22	0.17	29.50	0.258
N1	5.68	254.71	0.058	1.701	21.32	0.19	37.30	0.385
N1	5.82	263.41	0.061	1.716	21.36	0.20	37.03	0.385
N1	5.71	259.74	0.064	1.730	21.43	0.20	36.75	0.388
N2	7.71	225.79	0.068	1.812	27.05	0.20	38.91	0.567
N2	7.47	215.56	0.069	1.824	27.16	0.21	38.62	0.581
N2	7.52	237.97	0.070	1.895	27.15	0.22	38.37	0.561
N3	8.01	211.33	0.078	2.003	34.58	0.20	40.20	0.779
N3	8.12	224.75	0.075	1.912	34.70	0.21	39.90	0.787
N3	7.84	215.05	0.077	2.011	34.76	0.22	39.68	0.763
N4	5.19	259.20	0.065	1.697	18.10	0.18	35.66	0.572
N4	5.12	267.14	0.060	1.701	18.03	0.19	35.40	0.576
N4	5.04	268.22	0.063	1.684	18.36	0.20	35.09	0.604
N5	4.26	294.26	0.052	1.556	18.04	0.16	32.12	0.575
N5	4.00	298.08	0.049	1.542	17.30	0.17	31.90	0.558
N5	4.12	301.15	0.050	1.561	17.66	0.18	31.52	0.561
